# Basidiobolomycosis Mimicking Fistulizing Crohn’s Disease: A Case Report From Saudi Arabia

**DOI:** 10.7759/cureus.37981

**Published:** 2023-04-22

**Authors:** Yaser Meeralam, Hajar Alsulami, Anas M Aljoaid, Mohammed Khayat, Saad Zahrani, Mutaz Khairo, Salem Alotaibi

**Affiliations:** 1 Digestive and Liver Health Center, King Abdullah Medical City, Makkah, SAU; 2 Internal Medicine, Al-Noor Specialist Hospital, Makkah, SAU; 3 Radiology Department, King Abdullah Medical City, Makkah, SAU

**Keywords:** fungal infection, management, diagnosis, crohn’s disease, gib

## Abstract

Gastrointestinal basidiobolomycosis (GIB) is a rare, emerging fungal infection caused by Basidiobolus ranarum, which requires a high index of clinical suspicion for early diagnosis and management. It is prevalent in hot and humid regions, and its clinical manifestations may mimic inflammatory bowel disease (IBD), malignancy, and tuberculosis (TB). This often results in the disease being missed or incorrectly diagnosed.

We present the case of a 58-year-old female patient from the southern region of Saudi Arabia who presented with persistent non-bloody diarrhea for four weeks and was found to have GIB. This condition is associated with significant morbidity and mortality if not diagnosed and treated in a timely manner. The optimal therapeutic strategy for managing this rare infection has not yet been established. Most patients described in the literature have received a combination of pharmaceutical and surgical therapy. Including GIB in the differential diagnosis of gastrointestinal disorders that do not fit the diagnosis may help with its early diagnosis and management.

## Introduction

Basidiobolomycosis is a relatively uncommon infection caused by the fungus Basidiobolus ranarum, which belongs to the class Zygomycetes and the order Entomophthorales. It usually affects the skin and the tissue under the skin but can also affect other organs, including the gastrointestinal tract, lungs, and kidneys, among others [1]. Even though gastrointestinal basidiobolomycosis (GIB) is very rare, with only about 100 cases reported in the literature, its incidence is on the rise, with most of the cases reported in the last 20 years. GIB causes invasive infections in both immunocompromised and immunocompetent individuals. It is often misdiagnosed as carcinoma, inflammatory bowel disease (IBD), tuberculosis (TB), or a fungal infection like mucormycosis since it is quite rare and unfamiliar to the physician [2]. Owing to the rarity of the disease, its diagnosis and management are quite challenging. The nature of the infection can lead to fatal complications such as hepatic artery aneurysms, liver abscesses, and lung abscesses [3]. Furthermore, it can be misdiagnosed as other diseases and malignancies. Mohammadi et al. reported an unusual case of basidiobolomycosis, which was diagnosed initially as colon cancer [4]. Similarly, Vikram et al. reported the case of a 50-year-old male with a space-occupying lesion, which was diagnosed as cancer [2]. However, GIB can also be misdiagnosed as IBD, and such instances have been described in several studies in the literature [4-6]. The misdiagnosis is often caused by the nature of the disease, presenting with space-occupying lesions as well as with fistula and perforation [1-7].

Patients with GIB often have neutrophilic leukocytosis, a high erythrocyte sedimentation rate, and eosinophilia. Basidiobolomycosis can only be diagnosed by tissue culture, and when there is no culture available, a likely diagnosis can be made based on histopathology by the appearance of the tissue. Even though polymerase chain reaction (PCR) is not widely studied and available, a study in 2012 suggested the development of a specific PCR assay to detect the organism [8]. In most cases of GIB, the diagnosis is only made after surgery, based on histopathological findings like mixed suppurative and granulomatous inflammation, prominent eosinophilic infiltrates, and the presence of degenerate thin-walled broad hyphae surrounded by eosinophilic amorphous material, which is called the Splendore-Hoeppli phenomenon. The best way to manage GIB is with a combination of early surgery and the appropriate antifungal treatment. GIB is associated with significant mortality rates, reaching as high as 16% if left untreated [9]. We report a case of GIB in a female patient who had an entero-enteric fistula and colonic masses that mimicked Crohn’s disease.

## Case presentation

The patient was a 58-year-old female from the southern region of Saudi Arabia who was referred due to persistent non-bloody diarrhea for four weeks. She had visited the emergency department one month before with a complaint of non-bloody diarrhea and reported repeated incidents of vomiting food particles, which had started during the past five days and was associated with diffuse abdominal pain. Prior to that, she had been in her usual state of health. She denied night sweats, fever, loss of weight, or appetite. She had no family history of cancer, no past surgical history, and denied any recent travel. However, she had left leg deep venous thrombosis for which she was taking apixaban; she was also diabetic and anemic, and hence was on insulin and oral iron therapy. On physical examination, she appeared to be in good condition, and her vital signs were as follows: pulse: 106, respiratory rate 17, blood pressure 148/63, and temperature 36.4. Her abdomen was tender with a palpable firm mass in the lower right quadrant. The patient was admitted to the hospital and started on intravenous fluids for hydration and correction of hypokalemia (Table 1). The lab findings are presented in Table 1.

**Table 1:** Laboratory investigations CBC: complete blood count; WBC: white blood cells; RBC: red blood cells; HGB: hemoglobin; CRP: C-reactive protein; ESR: erythrocyte sedimentation rate; BUN: blood urea nitrogen

Test	Patient value	Reference range
CBC		
WBC	7.79 x 10^9^/L	3.9-11 x 10^9^/L
RBC	2.91 x 10^12^/L	4.3-5.7 x 10^12^/L
Eosinophils	0.79 x 10^9^/L	0.04-0.4 x 10^9^/L
HGB	7.9 gm/dl	12-15 gm/dl
Platelet	208 x 10^9^/L	150-400 x 10^9^/L
Alkaline phosphatases	633 U/L	46-118 U/L
Albumin	2.5 gm/dl	3.4-5 gm/dl
Alanine transaminase	28 U/L	12-78 U/L
Aspartate transaminase	26 U/L	8-43 U/L
Direct bilirubin	0.08 mg/dl	0-0.3 mg/dl
Total bilirubin	0.21 mg/dl	<1.2 mg/dl
Total protein	6.78 gm/dl	6.3-7.9 gm/dl
CRP	9.64 mg/dl	0-0.29 mg/dl
ESR	140 mm/h	0-20 mm/h
Potassium	2.35 mmol/L	3.5-5.1 mmol/L
Sodium	138 mmol/L	136-150 mmol/L
Bicarbonate	20 mmol/L	21-32 mmol/L
BUN	46.65 mg/dl	8-24 mg/dl
CA 125	84.9 U/ml	0-30.2 U/ml
Calprotectin	545 MG/L	<50 MG/L

**Figure 1:**
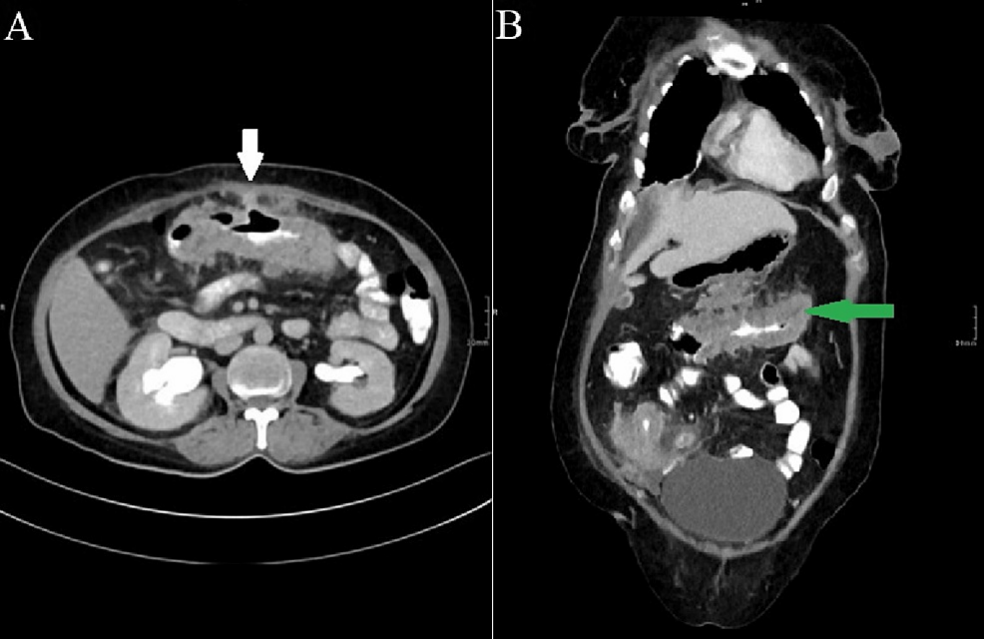
CT scan of the abdomen - (A) axial view showing circumferential mural wall thickening (white arrow). (B) Sagittal view showing circumferential mural wall thickening of the colon (green arrow)

**Figure 2:**
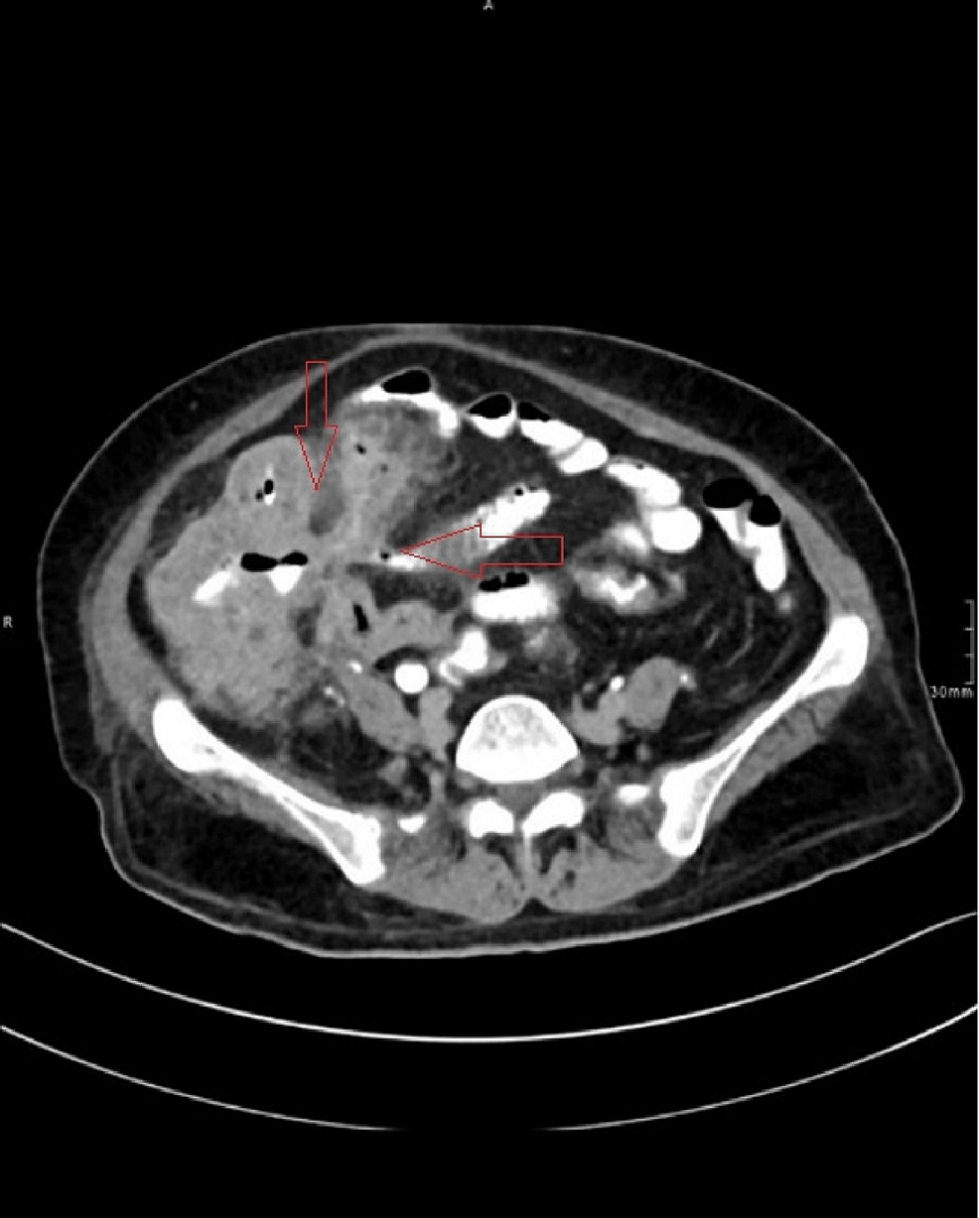
CT of the abdomen showing an entero-colonic fistula CT: computed tomography

The patient was referred for a contrast-enhanced CT scan of the abdomen and pelvis, which revealed inflammation of the intestine with the formation of multiple fistulas and soft tissue swelling in the mesentery extending to the anterior abdominal wall. The scan also showed a sealed perforation of the appendix with a collection (Figures 1-2).

**Figure 3:**
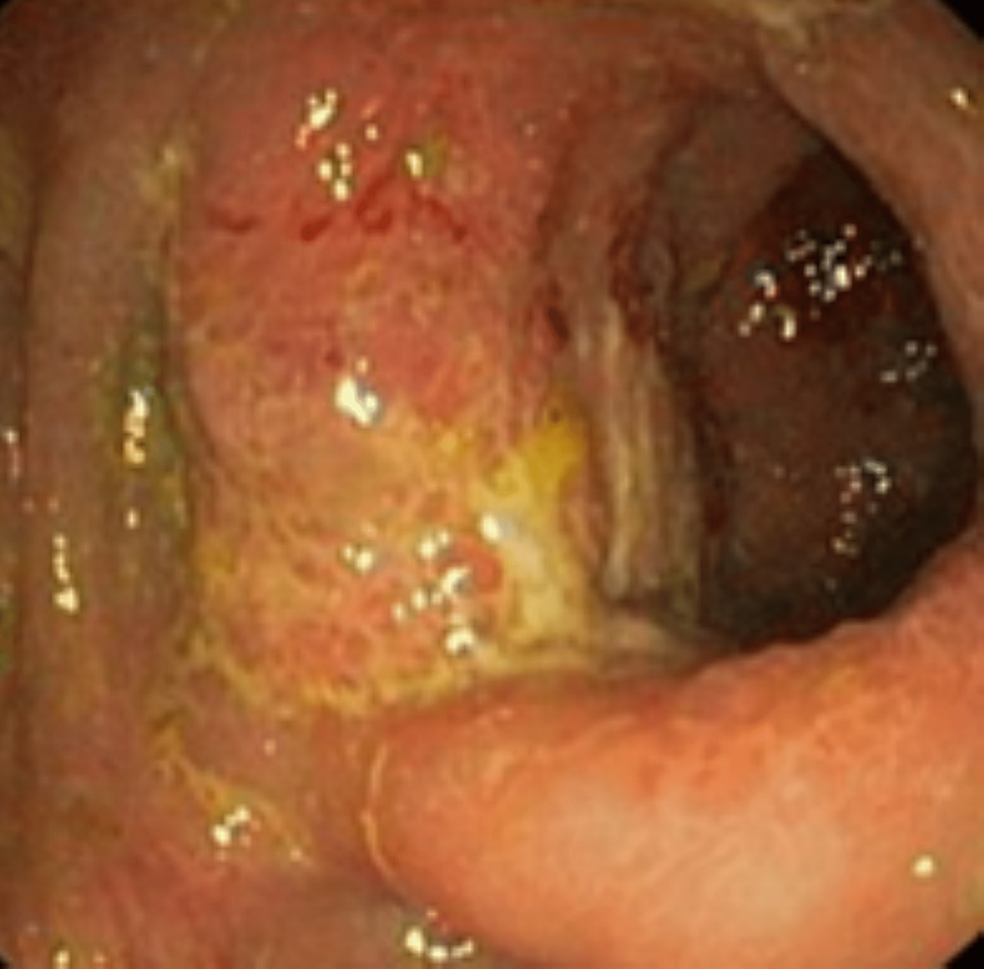
Colonoscopy showing multiple fungating masses spread throughout the colon

The patient then underwent esophagogastroduodenoscopy and colonoscopy. The esophagogastroduodenoscopy showed mild nonspecific esophagitis, while the colonoscopy revealed severe inflammation involving the proximal sigmoid colon with a deep ulcer, thick mucosa with narrowing, another polypoid circumferential mass involving the mid transverse colon with significant narrowing, and fragile mucosa with a possible fistula opening (Figure 3).

**Figure 4:**
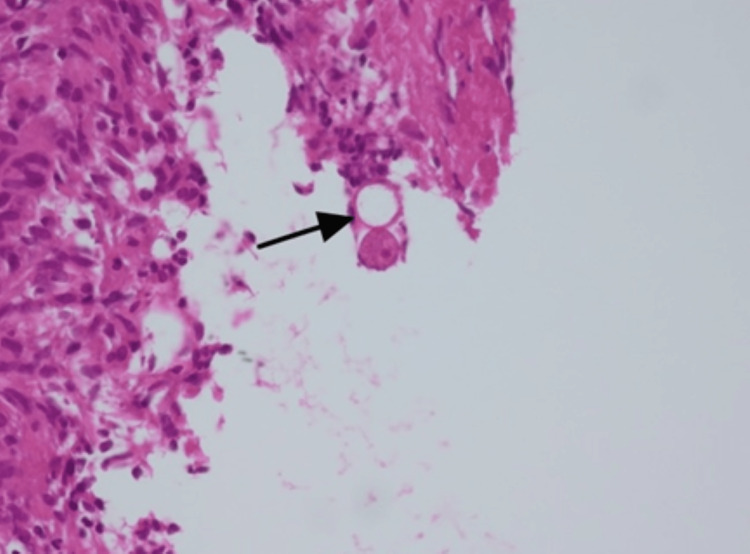
Histopathology (the arrow shows fungal spore)

The histopathology report of the patient showed two core biopsy segments of fibroadipose tissue exhibiting multiple granulomas composed of epithelioid cells and multinucleated giant cells and areas of inflammation with a predominance of eosinophils and fibrosis. Scattered, broad, sparsely septate fungal hyphae were present in the granulomas. In addition, fungal rounded zygospores with a thin outer wall, foamy cytoplasm, and nucleus were noted. The PAS and GMS stains highlighted these fungal organisms. No malignancy was seen in the examined biopsy material. Biopsies for TB PCR and culture and acid-fast bacilli (AFB) were negative. The morphologic features of fungal organisms were suggestive of basidiobolomycosis, ruling out the diagnosis of Crohn’s disease (Figure 4).

**Figure 5:**
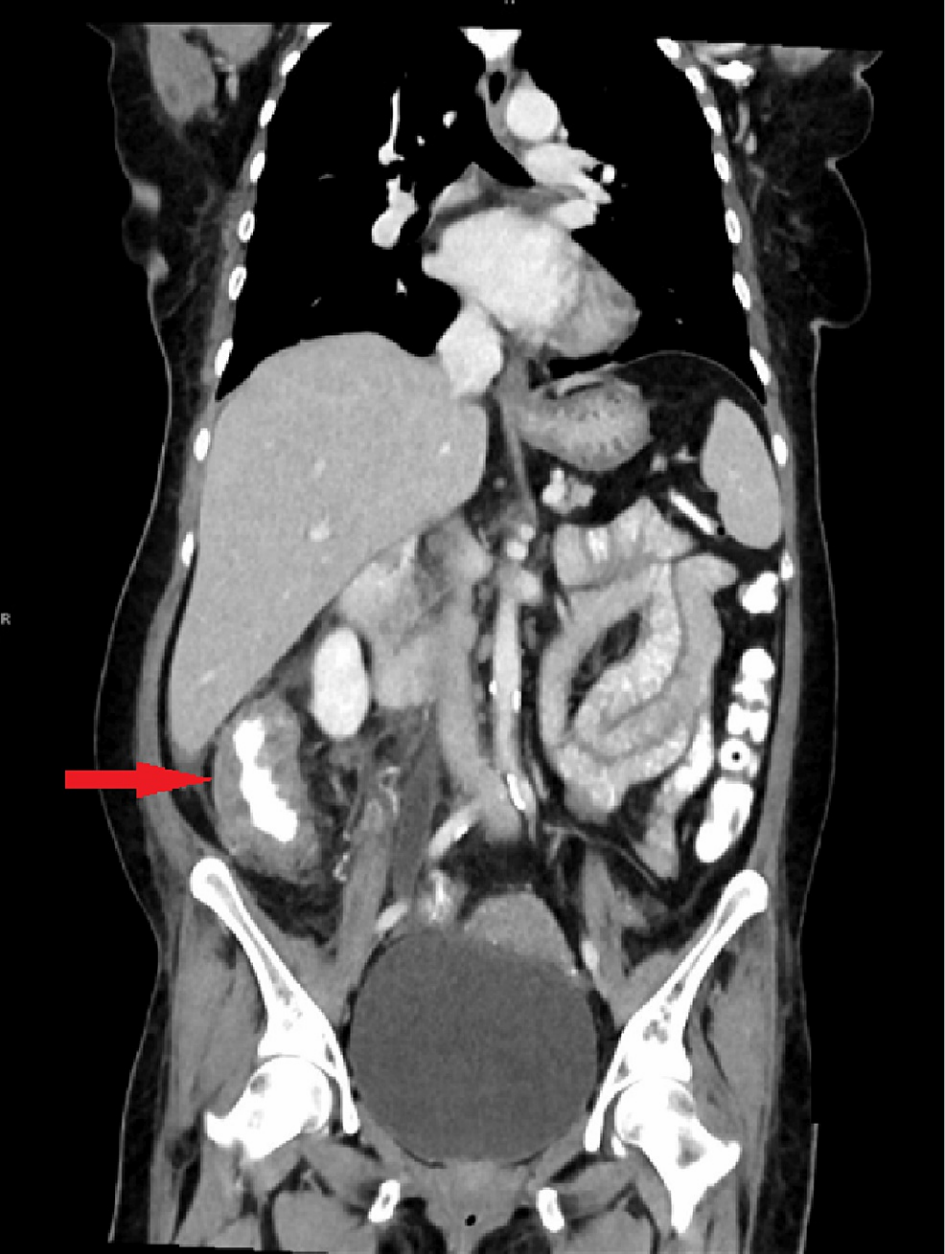
CT of the abdomen and pelvis showing perforated colon CT: computed tomography

The patient was advised to start voriconazole 200 mg orally every 12 hours for two weeks, but she could not tolerate it due to moderate non-bloody diarrhea, and hyperkalemia. She was then switched to itraconazole 200 mg once daily as per the recommendations from the infectious disease specialty, which resulted in a dramatic improvement in her diarrhea. At her two-week follow-up, the patient had no abdominal pain and her diarrhea had completely resolved with the itraconazole treatment. However, one month later, she presented to the emergency department with severe abdominal pain, guarding, and rigidity. She was then referred for an emergency CT abdomen and pelvic scan, which showed a perforated colon (Figure 5).

The patient was taken to the operating room for an exploratory laparotomy, during which a retroperitoneal cecal perforation with fecal matter and a pyogenic membrane was revealed. There was also thickening of the lumen in the ascending colon, hepatic flexure, and transverse colon, as well as severe adhesions between the transverse colon, omentum, stomach, and abdominal wall. Severe adhesion was also found between multiple loops of the small bowel and the right colon, but no entero-enteric fistula was found. Upon examining the small bowel, left colon, and rectum, no abnormalities were detected. The operation concluded with an extended right hemicolectomy and end ileostomy. The interventional radiology team drained the abdominal collection due to the extensive adhesion found during the laparotomy. The findings from radiology and histopathology confirmed the diagnosis of colon basidiobolomycosis.

## Discussion

GIB is an emerging infection that can only be diagnosed by maintaining a high level of suspicion. It is associated with significant morbidity and mortality if not diagnosed and treated early. Including GIB in the differential diagnosis of an abdominal mass with eosinophilia or cases that resemble IBD but do not fit the diagnosis may help in early diagnosis and management [6]. As GIB is a rare disease with nonspecific gastrointestinal symptoms, its clinical presentation can be easily confused with more common gastrointestinal diseases [5]. Differential diagnoses for GIB include Crohn's disease, intestinal TB, sarcoidosis, and amebiasis. Inflammation around the organs, fistulization, perforation, and abscess formation may resemble Crohn's disease [10]. This was true in our case as well since our patient initially presented symptoms suggestive of Crohn's disease. The first reported case of GIB dates back to 1964 when a four-year-old boy was diagnosed with the infection. Since then, cases of GIB have been reported from different parts of the world, including Saudi Arabia, Kuwait, the United States, and Iran. However, in all previous cases, GIB was not diagnosed until after surgery had been performed. The pathological diagnosis of GIB cases typically reveals the presence of Splendore-Hoeppli bodies, a high number of eosinophils, and granular eosinophilic material that radiates strongly around the fungal elements. This is a very typical histological finding for this fungus [11]. Eosinophils and changes are consistent with inflammation. All of the patients had high white blood cell counts with eosinophilia, which has been linked to systemic fungal infections like coccidioidomycosis but not often to gastrointestinal cancers or IBD [12]. The histological findings of our patient showed multiple granulomas with scattered, broad, sparsely septate fungal hyphae, in addition to fungal rounded zygospores and areas of inflammation with a predominance of eosinophils and fibrosis.

Optimal therapeutic strategies for the management of this rare infection have not yet been established. Most patients have received a combination of pharmaceutical and surgical therapy. Although there have been reports of patients showing improvement with antifungal therapy alone, the long-term results are still unknown. Itraconazole has been used to treat fungal infections in most cases (73%), followed by amphotericin (22%), ketoconazole (8%), and voriconazole (5%). Potassium iodide and trimethoprim/sulfamethoxazole may also be effective in clinical settings. Antifungal therapy has been administered for an average of eight months, and the overall survival rate is believed to be 80%. Notably, the use of amphotericin has been linked to clinical failures in several cases, and amphotericin resistance has been confirmed in some of these cases by susceptibility testing. In our case, the patient completed six months of antifungal therapy with a complete improvement of her clinical symptoms and without further complications. In addition, there was continuous monitoring during therapy for side effects but none were reported. Posaconazole could help treat this infection in several ways, as it is well-tolerated and has few side effects [13]. Antifungal and surgical treatments are considered to be the cornerstones of GIB treatment, but unfortunately, the disease has a mortality rate of 16% if not treated appropriately. The duration of antifungal treatment varies and depends on patient response, ranging from four months to one to two years, with an average of eight months [14].

Albishri et al. reported three cases of GIB who were also from the southern region of Saudi Arabia and were treated successfully with single oral itraconazole therapy and did not eventually need surgery [15]. Similarly, in another case report from Saudi Arabia, treatment with voriconazole alone for a year was successful in an 11-year-old boy, and there was full recovery and no recurrence of the infection after a year [16]. Likewise, another case of GIB in a 36-year-old Saudi Arabian male has been reported; he was successfully treated with voriconazole after previously being resistant to itraconazole [17]. Alabdan et al. reported another case of GIB in a 45-year-old Saudi Arabian woman who was successfully treated with itraconazole therapy and was symptom-free at the six-week follow-up [7]. In our case, voriconazole was started, but after a few days, the patient started to have refractory hyperkalemia, due to which she was switched to itraconazole. According to Choi et al., azole antifungals may interfere with the biosynthesis of adrenal steroids and therefore can predispose patients to aldosterone deficiency, which can lead to hyperkalemia [18]. Many reports have shown successful treatment of GIB with voriconazole alone without surgical resection [11-14]. However, this contrasts with our case, as our patient required immediate surgical intervention, which was performed in the form of an extended right hemicolectomy with end ileostomy due to perforation.

## Conclusions

Gastrointestinal basidiobolomycosis is an emerging infection that can lead to significant morbidity and mortality, and it presents considerable diagnostic challenges. Clinicians should be vigilant when encountering patients from tropical areas presenting with symptoms such as abdominal pain, masses, and diarrhea with peripheral eosinophilia or findings resembling IBD. Further research is required to better understand its etiology and develop effective management strategies.

## References

[1] Shaikh N, Hussain KA, Petraitiene R, Schuetz AN, Walsh TJ (2016). Entomophthoramycosis: a neglected tropical mycosis. Clin Microbiol Infect.

[2] Vs V, Hallur V, Samal S, Chouhan MI, Bhat SJ, Kumar P, Mishra TS (2021). Basidiobolomycosis of right colon mimicking as carcinoma of colon. ACG Case Rep J.

[3] Omar Takrouni A, Heitham Schammut M, Al-Otaibi M, Al-Mulla M, Privitera A (2019). Disseminated intestinal basidiobolomycosis with mycotic aneurysm mimicking obstructing colon cancer. BMJ Case Rep.

[4] Mohammadi R, Ansari Chaharsoghi M, Khorvash F (2019). An unusual case of gastrointestinal basidiobolomycosis mimicking colon cancer; literature and review. J Mycol Med.

[5] Al Asmi MM, Faqeehi HY, Alshahrani DA, Al-Hussaini AA (2013). A case of pediatric gastrointestinal basidiobolomycosis mimicking Crohn's disease. Saudi Med J.

[6] AlSaleem K, Al-Mehaidib A, Banemai M, bin-Hussain I, Faqih M, Al Mehmadi A (2013). Gastrointestinal basidiobolomycosis: mimicking Crohns disease case report and review of the literature. Ann Saudi Med.

[7] Alabdan L, Amer SM, Alnabi Z, Alhaddab N, Almustanyir S (2020). Gastrointestinal basidiobolomycosis in a 45-year-old woman. Cureus.

[8] Gómez-Muñoz MT, Fernández-Barredo S, Martínez-Díaz RA, Pérez-Gracia MT, Ponce-Gordo F (2012). Development of a specific polymerase chain reaction assay for the detection of Basidiobolus. Mycologia.

[9] Balkhair A, Al Wahaibi A, Al-Qadhi H, Al-Harthy A, Lakhtakia R, Rasool W, Ibrahim S (2019). Gastrointestinal basidiobolomycosis: beware of the great masquerade a case report. IDCases.

[10] Sawan AS, Mufti ST, Rawas I (2010). Basidiobolomycosis a case report. New Egypt J Med.

[11] Geramizadeh B, Heidari M, Shekarkhar G (2015). Gastrointestinal basidiobolomycosis, a rare and under-diagnosed fungal infection in immunocompetent hosts: a review article. Iran J Med Sci.

[12] Lyon GM, Smilack JD, Komatsu KK (2001). Gastrointestinal basidiobolomycosis in Arizona: clinical and epidemiological characteristics and review of the literature. Clin Infect Dis.

[13] Rose SR, Lindsley MD, Hurst SF, Paddock CD, Damodaran T, Bennett J (2012). Gastrointestinal basidiobolomycosis treated with posaconazole. Med Mycol Case Rep.

[14] Ejtehadi F, Anushiravani A, Bananzadeh A, Geramizadeh B (2014). Gastrointestinal basidiobolomycosis accompanied by liver involvement: a case report. Iran Red Crescent Med J.

[15] Albishri A, Hegazy M, Hader H (2020). Gastrointestinal basidiobolomycosis. J Pediatr Surg Case Rep.

[16] Albaradi BA, Babiker AM, Al-Qahtani HS (2014). Successful treatment of gastrointestinal basidiobolomycosis with voriconazole without surgical intervention. J Trop Pediatr.

[17] Al-Naemi AQ, Khan LA, Al-Naemi I, Amin K, Athlawy YA, Awad A, Sun Z (2015). A case report of gastrointestinal basidiobolomycosis treated with voriconazole: a rare emerging entity. Medicine (Baltimore).

[18] Choi JY, Cho SG, Jang KS, Kim GH (2020). Voriconazole-induced severe hyperkalemia precipitated by multiple drug interactions. Electrolyte Blood Press.

